# Association of psychological distress and widespread pain with sympatoms of temporomandibular disorders and self‐reported bruxism in students

**DOI:** 10.1002/cre2.472

**Published:** 2021-07-20

**Authors:** Outi S. Huhtela, Ritva Näpänkangas, Anna Liisa Suominen, Jaro Karppinen, Kristina Kunttu, Kirsi Sipilä

**Affiliations:** ^1^ University of Eastern Finland ‐ Kuopio campus, Institute of Dentistry Kuopio Finland; ^2^ Faculty of Medicine, Research Unit of Oral Health Sciences Oulu University Oulu Finland; ^3^ Medical Research Center, Oral and Maxillofacial Department Oulu University Hospital Oulu Finland; ^4^ Centre for Life Course Health Research, Faculty of Medicine University of Oulu Oulu Finland; ^5^ Occupational Health Finnish Institute of Occupational Health Helsinki Finland; ^6^ Finnish Student Health Service Turku Finland

**Keywords:** bruxism, psychosocial distress, student, temporomandibular disorder

## Abstract

**Objectives:**

The aim of this study was to evaluate the association of psychosocial distress and widespread pain with self‐reported symptoms of temporomandibular disorders (TMD) and bruxism, in two cross‐sectional surveys in 2012 and 2016, and whether there are temporal changes in the magnitude of associations.

**Materials and methods:**

The data were gathered from Finnish university students in 2012 and 2016. TMD symptoms were assessed with three validated questions and bruxism with one frequently used question. Psychosocial distress was assessed with the General Health Questionnaire‐12 (GHQ‐12), and widespread pain with questions of pain in the extremities, the neck or upper back, and lower back. The associations of GHQ‐12, widespread pain and background variables with TMD symptoms and bruxism were analyzed with chi‐square tests, t‐test and binary logistic regression models stratified by gender, and adjusted for age‐group, self‐reported general health/wellbeing and presence of widespread pain.

**Results:**

Higher GHQ‐12 score and presence of widespread pain were significantly associated with TMD symptoms in both genders at both time points. The association of higher GHQ‐12 score with sleep bruxism and awake bruxism were inconsistent. In the adjusted model higher GHQ‐12 score and widespread pain were significantly related to TMD pain symptoms in both genders at both time points, and to bruxism in 2012. Between the two time points a greater variability in these associations was seen in men than in women.

**Conclusions:**

Psychological distress and widespread pain are significant determinants in perceived TMD pain and bruxism among students. No significant temporal alterations were observed.

## 1 BACKGROUND

Temporomandibular disorders (TMD) is a term used for a complex variety of pain and disorders in the masticatory muscles and the temporomandibular joints (TMJs). The most common TMD symptoms include pain in masticatory muscles, TMD‐related headache, and restricted jaw function. TMD symptoms are most common in 20‐ to 50‐year olds in the general population, and women are twice as often affected as men (Bueno et al., 2018; LeResche, 1997; Maixner et al., 2011). TMD symptoms are prevalent especially in students (Huhtela et al., 2016; Miyake et al., 2004; Pedroni et al., 2003). In our previous study, 21% of Finnish students (26% of women and 11% of men) reported TMD symptoms, especially those related to pain (Huhtela et al., 2016).

The etiology of TMD is multifactorial and complex, including biopsychosocial, genetic, and environmental factors (Maixner et al., 2011). TMD can be included in musculoskeletal disorders and chronic pain disorders in general, and individuals with TMD often have overlapping pain symptoms in other areas of the body (Bair et al., 2016). It has been shown that TMD linked with multiple pain affects more women than men (Sipilä et al., 2011).

The biopsychosocial model of TMD comprises general health factors and other pain symptoms; bruxism, various psychological characteristics, like depression, anxiety, and catastrophizing; and social and genetic factors (Suvinen et al., 2005). The biopsychosocial paradigm in pain research is well accepted (Gatchel et al., 2007); TMD and other musculoskeletal pain share same multifactorial biopsychosocial background, including psychosocial factors such as anxiety, distress, and depression (Sipilä et al., 2006; Suvinen et al., 2004; Tuuliainen et al., 2015). According to the biopsychosocial model, the relationship between psychosocial factors and pain chronification is bidirectional (Edwards et al., 2016).

According to a recent definition, bruxism refers to “both diurnal repetitive or sustained tooth contact by bracing or thrusting of the mandible; and nocturnal rhythmic or non‐rhythmic masticatory muscle activities” (Lobbezoo et al., 2013; Lobbezoo et al., 2018). In our previous study, self‐reported bruxism occurred among 26% of the students and was strongly associated with TMD symptoms (Huhtela et al., 2016).

TMD symptoms, bruxism, and widespread pains are strongly associated with perceived distress in the general population (Glaros et al., 2005; Tuuliainen et al., 2015; Yap et al., 2002). Psychosocial distress is reflected by five attributes: perceived inability to cope effectively, change in emotional status, discomfort, communication of discomfort, and harm; where harm is a temporary or permanent negative individual response to a stressor (Ridner, 2004). Individual coping skills, among other antecedents, influence the manifestation of these attributes. The 12‐item General Health Questionnaire (GHQ‐12) applied in this study is a screening instrument for measuring coping resources, psychological distress, and minor psychiatric disorders, at the population level (Goldberg et al., 1997). Studies on the comorbidities of chronic pain conclude that active coping strategies have a positive impact on pain management, while passive coping methods, like avoidance, are regarded as maladaptive (Zakrzewska, 2013). Thus, people with psychologically passive coping abilities tend to be more susceptible to developing chronic pain.

Students entering a new period of life face both autonomy and new responsibilities. They report more psychological distress than the general population possibly linked with altered life situations, economic problems, pressure from education administrations, academic demands and living conditions (Deasy et al., 2014; Marshall et al., 2008; Stallman, 2010). In Finnish university students, the prevalence of psychological distress and the use of mental health services have increased during the past decade; psychological distress and depression, inter alia, prompt them to seek psychological or psychiatric help (Oksanen et al., 2014).

Like TMD, bruxism also shares partly the same background factors, such as psychosocial distress and anxiety (Aggarwal et al., 2010; Kuhn & Türp, 2018; Manfredini & Lobbezoo, 2009). Studies, with small sample sizes, have reported that bruxism may be associated with other musculoskeletal pain conditions (Baad‐Hansen et al., 2019; de Siqueira et al., 2017). However, the studies are scarce, and thus further studies are needed.

The aim of this study was to evaluate the association of psychosocial distress and widespread pain with self‐reported TMD symptoms and bruxism, in two cross‐sectional surveys in 2012 and 2016, and whether there are temporal changes in the magnitude of associations. We hypothesize that psychological distress and widespread pain are associated with both TMD symptoms and bruxism in students, and that women are more susceptible to multiple symptoms than men.

## 2 MATERIALS AND METHODS

### 2.1 Subjects

The data was derived from two analogous nationally representative postal questionnaire surveys, the Finnish University Student Health Survey 2012 and 2016, performed by the Finnish Student Health Service (FSHS). The target population in 2012 consisted of 276,279 and in 2016 of 208,825 undergraduate students, aged 18–34 years and studying in Finnish universities (academic universities and universities of applied sciences). Randomization was performed according to study location, and age group and gender were taken into account when the representativeness was evaluated. A more detailed information about the sampling procedures, description of the study population and respondents, and the randomization is presented elsewhere (Kunttu et al., 2017; Kunttu & Pesonen, 2013).

The samples in 2012 and 2016 consisted of 9992 and 10,000 students, of whom 47 and 48%, respectively, were men. The distributions of students in academic universities and universities of applied sciences were equal, and the gender distribution was equal in both surveys. The overall response rate was 44.1% (men 34.9%, women 52.1%) in 2012 and 30.8% (men 22.3%, women 38.6%) in 2016. The age range of the respondents was 19–34 years, the mean (*SD*) age being 24.9 (3.6) years in 2012 and 25.0 (3.4) years in 2016 (Kunttu et al., 2017; Kunttu & Pesonen, 2013).

### 2.2 Ethics

The study plan was approved in 2012 by the Medical Ethics Committee of the Hospital District of Southwest Finland, and in 2016 by the Ethics Committee of the University of Turku. Permission for the implementation of the study was given by the authorities of FSHS.

### 2.3 Data collection

A postal questionnaire in Finnish included 168 comprehensive questions on health, health‐related behavior, needs and use of health services, study ability and social relationships. The questionnaire could be filled online. Four reminders (either postal or online reminders/questionnaires) were sent to the non‐respondents in both studies. Altogether 4403 students returned their answers between February and September 2012, and 3082 students between February and May 2016 (Kunttu et al., 2017; Kunttu & Pesonen, 2013).

### 2.4 TMD symptoms and bruxism

The questionnaire included four questions on TMD symptoms and bruxism both in 2012 and 2016. The following three questions that have been shown to be valid for screening TMD pain and jaw locking (Lövgren et al., 2016) were used:

1. TMD pain: Do you experience pain in the temples, TMJ, face or jaw once a week or more often? (with the following answer options: no/yes, occasionally/yes, all the time)
2. TMD pain on jaw movement: Do you experience pain once a week or more often while opening your mouth wide open or during chewing? (never or seldom/yes),
3. TMJ locking: Does your jaw lock once a week or more often? (no/yes).
  Furthermore, the questionnaire included the following frequently used question concerning bruxism:
4. Sleep bruxism (SB)/awake bruxism (AB): Do you grind or clench your teeth tight together? (no/only when sleeping/only awake/both asleep and awake).

### 2.5 Psychological distress

The present study used the 12‐item General Health Questionnaire (GHQ‐12) as an instrument for assessment of psychological distress. The GHQ‐12 is a well‐known screening instrument to measure non‐specific psychiatric morbidity with 12 items, such as satisfaction with oneself and with one's life situation (Goldberg et al., 1997; Gouveia et al., 2010). The reliability of the GHQ‐12 has been verified previously (Oksanen et al., 2014), while Hankins et al. showed the validity of the scoring of GHQ‐12 (Hankins, 2008). Its validity has also been evaluated in the Finnish Health 2000 Survey (Aalto et al., 2012). The GHQ‐12 instrument includes questions regarding concentration, decision making, coping with difficulties, feelings of usefulness, happiness, self‐confidence, and sleep disturbances, for example. Respondents were asked to rate the extent to which they had recently experienced any of the 12 symptoms, using a 4‐point Likert scale (1 = not at all, 2 = same as usual, 3 = somewhat more than usual, and 4 = much more than usual). The responses were combined into a sum score. A higher score indicated greater distress, the range being 12–48. Two out of 12 questions could be missing and replaced by the mean value of the remaining GHQ‐12 items of the individual. In the present study, GHQ‐12 was used both as categorized and continuous.

### 2.6 General health

In 2012, self‐rated general health was inquired with the following question: “How would you rate your own health?” (with options good/quite good/average/quite poor/poor). For the analysis, the responses were classified in three subgroups: good (good/quite good), moderate (average), and poor (quite poor/poor).

In 2016, self‐rated well‐being was inquired with the following question: “How would you describe your current state of overall well‐being?” (with options very good/good/fairly good/poor/very poor). For the analysis, the responses were divided into three subgroups: good (very good/good), moderate (fairly good), and poor (poor/ very poor).

### 2.7 Widespread pain

Questions of other pain items were inquired with the question “Have you had the following symptoms during the last month (30 days)?: Headache, neck or upper back pain, lower back pain, and pain in limbs” (with options not at all/ occasionally/weekly/daily or almost daily). The responses were dichotomized as 0 (not at all/occasionally) and 1 (weekly/daily or almost daily). Widespread pain was defined based on pain report in neck, back, and limbs according to White et al. (1999) with some modifications; subjects that reported pain involving at least one extremity, and either the neck or back, were assessed to be suffering from widespread pain.

### 2.8 Statistical analysis

For the analyses, age was categorized into 18–25 and 26–35‐year olds and GHQ‐12 score in quartiles with cut‐offs at ≤20, 21–23, 24–27, ≥28 of sum scores. Independent samples T‐test was used to analyze differences in mean age and GHQ‐12 scores between men and women. Chi‐square tests were used to evaluate the associations of age group, GHQ‐12 quartile, self‐reported general health/wellbeing, other pain items, and widespread pain with gender and occurrence of TMD symptoms (TMD pain, pain in jaw movement, and locking) and bruxism (SB, AB, and having both SB and AB). Logistic regression models were used to evaluate the adjusted associations between distress (GHQ‐12 score) and occurrence of bruxism (reporting both AB and SB), TMD pain and TMD pain on jaw movement. Associations were adjusted for age‐group, self‐reported general health/wellbeing and presence of widespread pain. Separate models were run with GHQ‐12 as continuous and categorized in quartiles. The frequency of distress as dispersion from GHQ‐12 mean was calculated.

## 3 RESULTS

Descriptive characteristics of the study population, stratified by gender, are shown in Table 1. Almost 25% of the subjects showed highest level of distress at both time points, women having significantly higher distress than men.

**TABLE 1:** Description of the study populations

	2012							2016						
	Men (*n* = 1628)		Women (*n* = 2775)		Total (*n* = 4265)			Men (*n* = 1036)		Women (*n* = 1934)		Total (*n* = 2988)		
	Mean	*SD*	Mean	*SD*	Mean	*SD*	*p* Value*	Mean	*SD*	Mean	*SD*	Mean	*SD*	*p* Value*
Age (y)	24.9	3.6	24.2	3.6	24.5	3.6	0.000	25.2	3.4	24.8	3.5	25.0	3.4	0.000
GHQ‐12 score as continuous	23.0	5.2	24.7	5.8	24.1	5.7	0.000	23.6	5.5	24.8	6.2	24.4	6.0	0.000
	*n*	%	*n*	%	*n*	%	*p* Value**	*n*	%	*n*	%	*n*	%	*p* Value**
Age group (y)														
18–25	1031	63.3	1934	69.7	2965	67.3	0.000	589	58.1	1219	65.1	1811	62.6	0.001
26–35	597	36.7	841	30.3	1438	32.7		425	41.9	653	34.9	1080	37.4	
Self‐reported general health/wellbeing														
Good	1369	84.7	2302	83.4	3671	83.9	0.385	784	74.2	1469	73.3	2256	73.5	0.010
Moderate	199	12.3	379	13.7	578	13.2		216	20.4	458	22.9	679	22.1	
Poor	49	3.0	78	2.8	127	2.9		57	5.4	77	3.8	134	4.4	
GHQ‐12 quartiles														
Lowest	567	35.9	692	25.7	1259	29.5	0.000	339	32.7	518	26.8	858	28.8	0.000
Medium low	425	26.9	628	23.4	1053	24.7		286	27.6	470	24.3	756	25.4	
Medium high	348	22.1	642	23.9	990	23.2		195	18.8	428	22.1	624	21.0	
Highest	238	15.1	725	27.0	963	22.6		216	20.9	518	26.8	738	24.8	
GHQ‐12 score as continuous														
Under average	992	62.9	1712	63.7	2646	62.0		696	67.2	1246	64.4	1826	61.3	
Over average	586	37.1	975	36.3	1619	38.0		340	32.8	688	35.6	1150	38.7	
Other pain														
headache	1060	65.1	2375	85.6	3435	78.0	0.000	614	60.6	1413	75.3	2027	70.2	0.000
neck or upper back	777	47.7	2044	73.7	2821	64.1	0.000	454	47.1	1166	69.0	1620	61.1	0.000
lower back	672	41.3	1394	50.2	2066	46.9	0.000	383	39.7	891	50.1	1274	46.4	0.000
limbs	471	28.9	950	34.2	1421	32.3	0.000	280	30.0	617	34.8	897	33.2	0.037
Widespread pain	374	23.0	865	31.2	1239	28.1	0.000	191	22.2	402	27.8	593	25.7	0.011

*Note: p* Value* independent sample *T*‐test, *p* value** Chi square.

### 3.1 Prevalence of TMD symptoms and bruxism

Prevalence of TMD symptoms, and report of both AB and SB (AB+SB), SB, and AB, by age group, self‐reported general health/wellbeing, psychological distress, and pain in other areas including widespread pain are presented in Tables 2 and 3. The prevalence of TMD symptoms and bruxism were approximately at the same level at both time points.

**TABLE 2:** Association of age group, self‐reported general health/wellbeing, psychological distress measured by the General Health Questionnaire‐12 (GHQ‐12), other pain items, and widespread pain with symptoms of temporomandibular disorders (TMD)

	2012						2016					
	TMD pain		TMD pain on jaw movement		TMJ locking		TMD pain		TMD pain on jaw movement		TMJ locking	
	Men	Women	Men	Women	Men	Women	Men	Women	Men	Women	Men	Women
*n*	184	714	66	256	40	123	171	583	51	193	39	102
All (%)*	11.4	25.9	4.2	9.6	2.5	4.5	16.0	28.8	4.8	9.6	3.7	5.1
Age group (y)	%	%	%	%	%	%	%	%	%	%	%	%
18–25	10.0	24.8	3.9	8.7	2.4	4.5	14.9	28.5	5.2	8.8	4.2	5.9
26–35	14.0	28.5	4.7	11.4	2.8	4.6	18.8	34.8	5.0	12.2	3.6	4.6
*p* Value*	**0.015**	**0.039**	0.476	**0.028**	0.627	0.848	0.101	**0.005**	0.881	**0.020**	0.636	0.265
Self‐reported general health/wellbeing	%	%	%	%	%	%	%	%	%	%	%	%
Poor	22.4	50.6	8.3	17.1	2.0	9.1	42.1	45.5	14.3	14.5	7.1	3.9
Moderate	21.0	35.8	8.3	15.1	4.6	4.3	20.8	41.5	8.0	15.1	5.7	6.2
Good	9.7	23.4	3.5	8.4	2.2	4.4	12.8	24.0	3.3	7.7	2.8	4.9
*p* Value**	**<0.001**	**<0.001**	**0.003**	**<0.001**	0.145	0.145	**<0.001**	**<0.001**	**<0.001**	**<0.001**	0.053	0.487
GHQ‐12 quartiles	%	%	%	%	%	%	%	%	%	%	%	%
Lowest	6.8	16.4	2.0	6.4	0.7	2.8	10.0	20.8	2.7	6.0	2.1	4.5
Medium low	10.5	22.1	4.2	8.1	3.9	4.0	15.4	24.7	4.2	9.2	1.8	4.5
Medium high	13.1	28.9	4.8	10.3	1.8	6.0	18.5	31.5	4.1	11.5	4.7	5.2
Highest	21.2	36.9	7.8	13.7	5.1	5.3	24.5	38.0	9.9	11.0	8.0	5.2
*p* Value**	**<0.001**	< **0.001**	**0.003**	**<0.001**	**<0.001**	**0.028**	**<0.001**	**<0.001**	**0.001**	**0.014**	**0.001**	0.907
GHQ‐12 as continuous												
Mean (SE) no	22.72 (0.13)	24.08 (0.12)	22.93 (0.14)	24.56 (0.12)	22.97 (0.14)	24.66 (0.12)	23.23 (0.18)	24.22 (0.16)	23.42 (0.17)	24.71 (0.15)	23.46 (0.17)	24.83 (0.14)
Mean (SE) yes	25.25 (0.48)	26.51 (0.24)	25.68 (0.85)	26.45 (0.41)	25.24 (0.90)	26.03 (0.57)	25.70 (0.49)	26.38 (0.28)	27.32 (1.00)	26.31 (0.50)	27.66 (1.17)	25.23 (0.68)
*p* Value**	**<0.001**	**<0.001**	**<0.001**	**<0.001**	**0.009**	**0.013**	**<0.001**	**<0.001**	**<0.001**	**0.001**	**<0.001**	0.543
Other pain items	%	%	%	%	%	%	%	%	%	%	%	%
Headache												
Yes	15.4	28.5	5.1	10.4	2.9	4.8	20.4	32.8	5.6	10.3	4.5	5.3
No	4.2	10.3	2.6	4.4	1.8	3.1	7.8	9.7	2.8	3.7	2.0	3.7
*p* Value**	**<0.001**	**<0.001**	**0.018**	**<0.001**	**0.186**	**0.137**	**<0.001**	**<0.001**	**0.036**	**<0.001**	**0.039**	0.168
Upper back/neck												
Yes	17.6	30.3	6.1	16.1	3.0	5.1	20.7	30.4	5.1	10.0	6.2	5.6
No	5.8	13.6	2.6	6.0	2.0	2.9	8.3	13.7	2.6	4.1	1.6	3.7
*p* Value**	**<0.001**	**<0.001**	**0.001**	**<0.001**	**0.199**	**0.016**	**<0.001**	**<0.001**	**0.041**	**<0.001**	**<0.001**	0.090
Lower back												
Yes	16.6	32.1	8.9	11.3	3.4	5.1	22.2	37.5	6.8	13.6	5.3	6.4
No	7.8	19.6	3.8	4.5	1.9	4.0	11.0	20.7	3.1	5.5	2.8	4.4
*p* Value**	**<0.001**	**<0.001**	**0.001**	**<0.001**	0.074	0.184	**<0.001**	**<0.001**	**0.007**	**<0.001**	**0.044**	0.060
Limbs												
Yes	19.1	36.5	7.5	15.9	3.3	5.3	21.8	39.2	6.5	14.3	3.6	7.0
No	8.3	20.4	2.9	6.2	2.2	4.1	12.7	26.1	3.4	7.6	3.7	4.4
*p* Value**	**<0.001**	**<0.001**	**<0.001**	**<0.001**	0.226	0.172	**<0.001**	**<0.001**	**0.031**	**<0.001**	0.166	**0.001**
Widespread pain												
Yes	22.2	38.1	8.1	16.6	3.6	5.3	20.9	37.6	11.9	12.0	4.2	7.3
No	8.2	20.3	3.1	6.3	2.2	4.2	11.8	22.4	3.0	6.9	3.5	4.4
*p* Value**	**<0.001**	**<0.001**	**0.002**	**<0.001**	0.149	0.630	**0.001**	**<0.001**	0.235	**<0.001**	0.630	**0.029**

*Note*: *Independent sample *T*‐test, **Chi square; percentages describe the prevalence of each symptom by gender. Significance bolded, *p*<0.05.

**TABLE 3:** Association of age group, self‐reported general health/wellbeing, psychological distress measured by the General Health Questionnaire‐12 (GHQ‐12), other pain items, and widespread pain with bruxism (AB + SB), sleep bruxism (SB), and awake bruxism (AB)

	2012						2016					
	AB+SB		SB		AB		AB+SB		SB		AB	
	Men	Women	Men	Women	Men	Women	Men	Women	Men	Women	Men	Women
*n*	301	837	204	582	45	55	198	526	129	349	33	43
All (%)*	18.8	30.7	12.8	21.3	2.8	2.0	18.8	26.3	12.3	17.4	3.1	2.1
Age group (y)	%	%	%	%	%	%	%	%	%	%	%	%
18–25	17.0	28.5	11.3	20.4	3.1	2.0	18.1	25.5	11.7	16.8	3.4	2.5
26–35	22.0	35.7	15.3	23.5	2.2	1.9	22.0	33.6	14.7	22.5	3.1	1.7
*p* Value*	**0.015**	**<0.001**	**0.023**	0.072	0.284	0.845	0.126	**<0.001**	0.162	**0.003**	0.766	0.232
Self‐reported general health/wellbeing	%	%	%	%	%	%	%	%	%	%	%	%
Poor	22.4	46.2	16.3	25.6	2.0	3.8	30.4	37.3	16.1	18.7	5.3	0
Moderate	22.3	33.5	14.7	20.1	3.0	2.9	21.0	32.0	10.0	19.3	3.7	2.6
Good	18.1	29.6	12.3	21.4	2.8	1.8	17.4	23.8	12.5	16.9	2.8	2.0
*p* Value**	0.289	**0.003**	0.469	0.549	0.926	0.155	**0.038**	**<0.001**	0.407	0.497	0.506	0.301
GHQ‐12 quartiles	%	%	%	%	%	%	%	%	%	%	%	%
Lowest	13.5	27.5	10.3	22.0	1.8	1.0	16.7	20.4	11.6	14.5	2.7	2.5
Medium low	20.2	28.9	14.2	20.5	2.9	1.8	20.8	24.5	14.4	15.9	3.8	1.9
Medium high	19.8	31.5	11.6	21.5	3.8	1.8	17.8	28.6	10.5	20.2	4.1	2.1
Highest	28.0	35.4	17.8	22.1	4.2	3.5	19.8	31.7	10.8	19.4	2.3	2.3
*p* Value**	**<0.001**	**0.008**	**0.022**	0.890	0.186	**0.009**	0.574	**<0.001**	0.507	0.059	0.620	0.930
GHQ‐12 as continuous												
Mean (SE) no	22.73 (0.14)	24.47 (0.13)	22.89 (0.14)	24.73 (0.13)	23.00 (0.14)	24.67 (0.11)	23.53 (0.19)	24.49 (0.16)	23.64 (0.19)	24.69 (0.16)	23.65 (0.18	24.84 (0.14)
Mean (SE) yes	24.31 (0.34)	25.28 (0.21)	23.96 (0.41)	24.68 (0.24)	24.00 (0.71)	27.44 (0.93)	23.99 (0.40)	25.92 (0.29)	23.41 (0.46)	25.67 (0.35)	23.24 (0.82)	24.95 (1.03)
*p* Value**	**<0.001**	**0.001**	**0.007**	0.861	0.207	**0.001**	0.274	**<0.001**	0.765	0.009	0.807	0.903
Other pain items	%	%	%	%	%	%	%	%	%	%	%	%
Headache												
Yes	21.4	32.2	14.3	22.4	3.4	2.0	22.4	29.1	15.2	19.6	3.8	2.1
No	14.0	21.8	9.9	14.9	1.8	2.0	13.7	15.2	8.7	10.2	2.3	2.4
*p* Value**	**<0.001**	**<0.001**	**0.011**	**0.001**	**0.070**	0.987	**0.001**	**<0.001**	**0.002**	**<0.001**	0.186	0.747
Upper back or neck												
Yes	23.6	32.4	15.6	22.0	3.4	2.2	23.7	28.0	16.1	19.1	3.1	2.1
No	14.5	25.9	10.2	19.5	2.3	1.4	13.7	17.0	8.9	12.2	2.9	1.9
*p* Value**	**<0.001**	**0.001**	**0.001**	**0.165**	**0.168**	0.167	**<0.001**	**<0.001**	**0.001**	**0.001**	0.901	0.839
Lower back												
Yes	22.3	34.5	14.5	23.2	2.9	2.6	24.8	32.8	14.7	21.2	5.7	2.9
No	16.4	26.8	11.5	19.4	2.8	1.5	15.1	21.2	11.3	15.2	1.5	1.2
*p* Value**	**0.003**	**<0.001**	0.081	**0.014**	0.862	0.405	**<0.001**	**<0.001**	0.122	**0.001**	**<0.001**	**0.013**
Limbs												
Yes	25.3	35.3	17.3	23.4	3.9	2.5	23.6	32.3	16.0	20.7	3.9	3.1
No	16.2	28.2	10.9	20.2	2.4	1.8	17.8	26.1	11.6	18.0	3.1	1.7
*p* Value**	**<0.001**	**<0.001**	**0.001**	**0.047**	0.098	0.216	0.076	**0.043**	0.458	0.244	0.219	0.433
Widespread pain												
Yes	27.3	36.2	18.0	23.7	4.4	2.7	24.5	31.3	16.0	21.0	4.7	3.2
No	16.3	28.2	11.2	20.3	2.4	1.7	17.6	24.5	12.1	17.2	2.8	1.6
*p* Value**	**<0.001**	**<0.001**	**0.001**	**0.045**	**0.040**	0.354	**0.036**	**0.009**	0.160	0.095	0.196	0.055

*Note*: *Independent sample *T*‐test, **Chi square. Significance bolded, *p*<0.05.

### 3.2 Associations between TMD symptoms, bruxism, age, and general health

Women aged 26–35 years were more likely to report AB+SB and TMD pain symptoms, as compared to women in the younger age group. Poor self‐rated general health was significantly associated with all TMD pain symptoms, and with AB+SB in 2012 and 2016, except for AB+SB among men in 2012.

### 3.3 Associations between TMD symptoms, bruxism, and distress

Among women with the highest distress level, the presence of TMD symptoms (except for TMJ locking in 2016) was approximately two‐fold as compared to those with the lowest level of distress. The same tendency was noted among men. The associations were significant in both genders at both time points, except for TMJ locking among women in 2016. The same associations were found when using GHQ‐12 as a continuous variable (Table 2).

In 2012, high distress level was significantly associated with AB+SB and AB among women and with AB+SB and SB among men. In 2016, high distress level was associated significantly with AB+SB and SB among women. When analyzing the associations with GHQ‐12 as a continuous variable, the associations with AB+SB were significant, whereas the associations with SB and AB varied (Table 3).

### 3.4 Associations between TMD symptoms, bruxism, and other pains

The associations of TMD symptoms and AB+SB with other pain items (headache, pain in neck or upper back, pain in lower back, and pain in limbs) are presented in Tables 2 and 3. Reporting headache and pain in other body sites, and widespread pain was associated significantly with TMD symptoms, except for TMJ locking, and with AB+SB.

In the logistic regression analysis when using general health as a categorical variable, GHQ‐12 as a continuous variable, and widespread pain as an independent variable, higher distress was associated significantly with TMD pain symptoms and AB+SB in both genders in 2012, and in 2016 with TMD pain symptoms and AB+SB only in women (Table 4). Widespread pain associated significantly with TMD pain symptoms at both time points, and with AB+SB in 2012 (Table 4). Between the two time points a greater variability in these associations was seen in men than in women: in men, the association between widespread pain and bruxism was weaker and between widespread pain and TMD pain symptoms stronger in 2016 as compared to 2012 (Table 4).

**TABLE 4:** Associations of psychosocial distress as measured with GHQ‐12, and co‐factors with bruxism (report of both sleep and awake bruxism), TMD pain and TMD pain on jaw movement as described by odds ratio (OR) and 95% confidence intervals (95%CI)

	Bruxism				TMD pain				TMD pain on jaw movement			
	2012		2016		2012		2016		2012		2016	
	Men OR (95% CI)	Women OR (95% CI)	Men OR (95% CI)	Women OR (95% CI)	Men OR (95% CI)	Women OR (95% CI)	Men OR (95% CI)	Women OR (95% CI)	Men OR (95% CI)	Women OR (95% CI)	Men OR (95% CI)	Women OR (95% CI)
Age group (y)												
18–25	Ref	Ref	Ref	Ref	Ref	Ref	Ref	Ref	Ref	Ref	Ref	Ref
26–35	1.24 (0.87–1.78)	1.22 (0.94–1.58)	1.24 (0.86–1.78)	1.22 (0.94–1.58)	1.24 (0.82–1.86)	1.17 (0.91–1.52)	1.24 (0.82–1.86)	1.17 (0.91–1.52)	0.65 (0.29–1.44)	0.64 (0.36–1.13)	0.47 (0.20–1.10)	1.31 (0.87–1.96)
*p* Value**	**0.015**	**<0.001**	0.126	**<0.001**	**0.013**	**0.039**	0.101	0.**005**	0.476	**0.028**	0.881	**0.020**
Self‐reported general health /wellbeing												
Good	Ref	Ref	Ref	Ref	Ref	Ref	Ref	Ref	Ref	Ref	Ref	Ref
Moderate	1.78 (0.80–3.97)	0.99 (0.49–2.00)	0.66 (0.29–1.49)	1.29 (0.64–2.61)	3.37 (1.56–7.29)	1.45 (0.75–2.84)	0.35 (0.16–0.77)	1.08 (0.55–2.12)	1.11 (0.26–4.85)	0.436 (0.06–3.38)	0.67 (0.16–2.91)	1.59 (0.53–4.76)
Poor	1.17 (0.73–1.88)	1.27 (0.93–1.74)	0.56 (0.25–1.25)	1.01 (0.50–2.04)	1.19 (0.70–2.01)	1.57 (1.16–2.14)	0.30 (0.14–0.64)	0.69 (0.35–1.34)	0.92 (0.35–2.41)	1.31 (0.72–2.41)	0.59 (0.13–2.57)	0.92 (0.30–2.77)
*p* Value **	0.289	**0.003**	**0.038**	**<0.001**	**<0.001**	**<0.001**	**<0.001**	**<0.001**	**0.003**	**<0.001**	**<0.001**	**<0.001**
GHQ‐12 continuous												
	0.99 (0.96–1.03)	1.02 (1.00–1.05)	0.99 (0.96–1.03)	1.02 (1.00–1.05)	1.04 (1.00–1.08)	1.03 (1.00–1.05)	1.04 (1.00–1.08)	1.03 (1.00–1.05)	1.09 (1.03–1.16)	0.99 (0.95–1.04)	1.06 (1.00–1.14)	1.02 (0.98–1.05)
*p* Value **	**<0.001**	**0.008**	0.574	**<0.001**	**<0.001**	**<0.001**	**<0.001**	**<0.001**	**0.003**	**<0.001**	**0.001**	**0.014**
Widespread pain												
	1.49 (1.00–2.24)	1.30 (0.99–1.71)	1.49 (1.00–2.24)	1.30 (0.99–1.71)	1.89 (1.21–2.95)	1.99 (1.52–2.59)	1.89 (1.21–2.95)	1.99 (1.52–2.59)	1.53 (0.65–3.60)	1.78 (1.07–2.98)	1.64 (0.70–3.86)	1.48 (0.98–2.24)
*p* Value **	**<0.001**	**<0.001**	**0.036**	**0.009**	**<0.001**	**<0.001**	**0.001**	**<0.001**	**<0.001**	**<0.001**	0.235	**0.002**

*Note*: Fitted separately for men and women. **Chi square test between genders. Significance bolded, *p*<0.05.

## 4 DISCUSSION

The present study was part of a comprehensive national questionnaire study on student population, using validated TMD questions and commonly used screening questions for bruxism. The results show both distress and widespread pain to be associated with TMD pain symptoms and, although less significantly, with bruxism, which at least partly supported the hypotheses.

### 4.1 Association between TMD and distress

Both distress and widespread pain were significantly associated with TMD pain symptoms, but not with TMJ locking, among both genders in 2012 and 2016. This corroborates previous studies showing the role of distress in TMD symptoms (Lei et al., 2016; Tuuliainen et al., 2015). Another Finnish study by Tuuliainen et al. (2015) investigated the association between TMD signs and psychological distress, using GHQ‐12, in a representative adult population study (*n* = 6155). Similar to the present study, GHQ‐12 was assessed both as a classified and continuous variable. They showed an association of high distress with masticatory muscle pain on palpation, TMJ pain on palpation in women, and TMJ crepitation in men. Additionally, Lei et al. (2016) studied the association between TMD symptoms and psychological distress, and sleep disturbances in a Chinese adolescent population (age 12–18 years, *n* = 620). TMD symptoms were inquired using the DC/TMD Symptom Questionnaire and distress with the Pittsburgh Sleep Quality Index and Depression, Anxiety, and Stress Scales 21. They showed that TMD symptoms associated significantly with psychological distress, sleep disturbances and daytime dysfunction, thus supporting the present results (Lei et al., 2016).

### 4.2 Association between TMD and general health and other pains

In the present study, widespread pain was the dominant determinant of TMD pain. The association between widespread pain and TMD pain symptoms found here supports the current conception of similarity between muscular orofacial pain and musculoskeletal pain (John et al., 2003). An earlier Finnish population‐based study (Health 2000 Survey, *n* = 6227) analyzed distinct pain‐profile clusters and showed that 5.8% of the subjects were included in the TMD‐linked multiple pain cluster. Furthermore, female gender, poor health, and chronic illnesses increased the probability of having both TMD and multiple pain (Sipilä et al., 2011). In the present study, however, the role of poor self‐reported health was nonsignificant, and the association between TMD and widespread pain was noted in both genders. Adding widespread pain to the multivariate model further decreased the strength of both general health and distress in the associations. This emphasizes the role of widespread pain in TMD pain as an independent determinant, besides distress, in this population. The differences between these two national surveys may, however, be explained by the selected samples. Although the population mainly comprised healthy young adults, with distress factors differing from the general population, the present study affirms our hypothesis of women being more susceptible to multiple symptoms than men. The presence of other pain symptoms and widespread pain in women exceeds that of men in almost all TMD symptoms and bruxism, which cannot be discarded despite the suggestion that women are more susceptible to reporting pain than men are. There was a remarkable increase in prevalence of pain symptoms between 2012 and 2016. One explanation for increasing the pain symptoms may be partly linked with the decrease in response rate from 2012 to 2016. Those who had more symptoms or problems, responded more to the questionnaire (Kunttu et al., 2017). Additionally, there is an actual increasing trend in the prevalence of frequent musculoskeletal pain over the period of 12 years among Finnish university students (Oksanen et al., 2014).

It is acknowledged that acute TMD may turn chronic with simultaneously existing widespread pain, with psychological factors affecting the individual's perception of distress and coping ability. Coping and sense of control of perceived distress also act as protective factors when pain is acknowledged. In an Australian population‐based study, high distress was associated with high prevalence of TMD pain in females, and elevated pain was better explained by lower perception of control than greater perception of distress (Sanders & Slade, 2011). However, the coping theory was not examined in the present study due to a lack of data on the chronicity and intensity of TMD pain and on coping. Additionally, based on the cross‐sectional study design, no conclusions about the direction of the relationships can be drawn.

### 4.3 Association between bruxism and distress

Based on the present study, the association of distress with bruxism was weaker than with TMD symptoms. A significant association between psychological distress and bruxism was shown in both men and women in 2012, whereas in 2016 the association was significant only in women. In this study, the associations of psychological distress with SB and AB were inconsistent by gender and time point, which corroborates the study of Cavallo et al. (2016). They studied the correlation of AB and SB with perceived stress in students using item eight from Fonseca's Questionnaire and the Perceived Stress Scale (PSS), and showed that although the association of perceived stress with both AB and SB was higher in women, a significant association between stress and AB was shown only in men (Cavallo et al., 2016). Saczuk et al. (2019) used PSS for stress, the Brief‐COPE‐scale for coping, and additionally a portable electromyogram/electrocardiogram device. They compared a group with symptoms of bruxism to an asymptomatic control group, and showed a relationship between distress and SB, and a gender variation between different coping strategies and SB (Saczuk et al., 2019).

A significant difference in association between distress and bruxism subscales SB, AB, and SB + AB is shown also by Bayar on dental patients (Bayar et al., 2012), of which those reporting SB + AB had distress more often than others, thus supporting the present results. However, in the present study some associations between distress and SB and AB were significant, but the low number of subjects in the subclasses decreases the power of the data and thus should be considered with caution. It should be noted that in the present study bruxism was inquired using a simple question, which gave no information on the grading or frequency of bruxism. Studies clarifying the definition and grading of sleep and awake bruxism are needed (Lobbezoo et al., 2018). Studies show that the mechanisms, etiology and physiopathology of sleep and awake bruxism are different and are possibly influenced, for example, by the person's chronotype (Serra‐Negra et al., 2017; van Selms et al., 2013). The variation in association between distress and sleep bruxism, awake bruxism, and overall bruxism found in the present study confirm the need for further studies.

The associations between bruxism, TMD pain symptoms and psychosocial background factors.

In the present study, widespread pain was strongly associated with both bruxism and TMD pain symptoms. Presence of widespread pain seemed to be a more significant explanatory factor for both TMD symptoms and bruxism, as compared to distress, the association of which weakened after adding widespread pain to the model. Earlier studies of the association between widespread pain and bruxism are scarce. In a single‐night polysomnographic study of sleep events on SB and TMD patients, those having also widespread pain showed less sleep efficiency than those without widespread pain (de Siqueira et al., 2017). Thus, the effect of widespread pain may be mediated through poor sleep quality, for example. It has been shown that muscle tension is high in widespread pain, headache and musculoskeletal pain in limbs, and in the upper and lower back (Wieckiewicz et al., 2014). This can be explained by the effect of somatic and/or mental stressors on muscle tension through the gamma‐loop, as described by Wieckiewicz et al. (2014). However, notwithstanding its limitations as a questionnaire study, this study indicates a confounding effect of other pain sites on the role of distress in TMD and bruxism. It has been suggested that distress is not a risk factor but a marker for onset of chronic pain (Aggarwal et al., 2010) and thus an unpredictable but manageable variable in tailored pain treatment. Prolonged pain experience and increased distress may sensitize the individual to pain in general; this connection may be linked with neurophysiological alterations in the central nervous system (Maixner et al., 2016; Yap et al., 2002).

In the two age groups, the present study found significant variation in self‐reporting of TMD symptoms and bruxism, mainly in women, whereas across symptoms some increase was seen. The most significant rise was seen in both groups in TMD pain, and respectively in bruxism in the older age group. There were some temporal changes in the associations of distress and widespread pain with the outcome variables. The association of high distress and widespread pain with bruxism weakened from 2012 to 2016, whereas the associations with TMD pain remained significant, or were stronger in 2016, as compared to the earlier data collection. The data did not offer any means for analyzing the possible background reasons for these variations; awareness may increase reporting of pain, whereas increased knowledge of attrition due to bruxism may motivate procurement of protective dental appliances, such as splints. These topics will be discussed in future studies.

### 4.4 Methodological considerations

A strength of the present study is the two large samples at two time points with similar questionnaires. It should be noted that the response rates were fairly low (44% in 2012 and 31% in 2016) which may affect the outcomes. However, except for the low male response rate, the respondents represented well the target population for the background variables (Kunttu & Pesonen, 2013), even if the percentage of all respondents was low in the 2016 study. Kunttu et al. conceded that the declining response activity may lead to a selected respondent group of those who are most troubled, which, however, is less detrimental than a situation where health problems would remain unidentified (Kunttu et al., 2017).

Another strength of the present study is the use of valid questionnaires for the assessment of TMD symptoms and psychological distress. GHQ‐12 is highly recommended for population‐based studies, while DC/TMD Axis II instruments are recommended for clinical studies on TMD patients (Ohrbach et al., 2010). In the present study, GHQ‐12 was used both as a categorized and continuous variable; the associations of distress with TMD symptoms and bruxism were compatible when using both approaches. However, it must be noted that the data are based on self‐reports. The three subclasses (AB, SB, AB+SB) of bruxism were inquired based on only one question, thus its occurrence must be considered only as probable, which may weaken this study. For practical reasons, the highest diagnostic level of bruxism, based on polysomnography is virtually impossible for large samples. Some studies have suggested that self‐report studies overestimate the prevalence of bruxism (Lobbezoo et al., 2013) which may also have affected on the associations found in the present study.

The role of psychological distress in TMD symptoms found in the present study indicates a psychosocial approach to the treatment of TMD. Some studies have shown a worsening impact of psychological factors on TMD treatment outcome (Huttunen et al., 2019; Sanders et al., 2016). In our previous study the applied relaxation (AR) method relieved muscular TMD pain more than splint treatment did, whereas the treatment responses on TMJ‐related findings were poorer (Huhtela et al., 2020). Interestingly, the subjects in the AR group also reported a decrease in other pains and psychosocial symptoms. According to our previous and present study, the TMD pain patient should be considered holistically due to possible underlying psychological burden and widespread pain. As most individuals with TMD pain have additional pain complaints in other regions of the body and are at a greater risk for associated somatic symptoms burden (Türp et al., 2016), assessment of the whole‐body pain using a pain drawing as well as a screening questionnaire for psychosocial assessment, using DC/TMD Axis II instruments are important for individual treatment planning (Hirsch & Türp, 2010).

A comprehensive tailored treatment plan might be essential to achieve best treatment outcome, although clinical studies are scarce and further studies on tailored treatment in TMD are needed.

The present study population was born and raised in the atmosphere of the 1990s economic challenges, which are known to enhance insecurity and distress for parents and thus indirectly their children's welfare. Linking psychological distress, parafunctional habits, and pain symptoms in the present study has only touched the surface. However, the notable increase in psychological distress and widespread pain in association with TMD pain symptoms seen in the present study is interesting and needs more well‐designed longitudinal studies.

## 5 CONCLUSION

Psychological distress and widespread pain are significant determinants in perceived TMD pain and self‐reported bruxism in students, and thus should be considered as important background factors. The impact of treatment of distress on TMD as well as bruxism needs to be studied further in clinical studies.

## CONFLICT OF INTEREST

The authors declare there is no conflict of interest.

## AUTHOR CONTRIBUTIONS

Outi S. Huhtela developed the study concept, performed the data analysis, and drafted the manuscript including the revisions. Ritva Näpänkangas aided in the manuscript preparation, Anna Liisa Suominen designed and supervised the data analysis and contributed to the manuscript preparation. Jaro Karppinen aided in the manuscript preparation. Kristina Kunttu designed the original study and contributed to the manuscript preparation. Kirsi Sipilä supervised the development of study concept, drafting of the manuscript, and provided the revisions to the manuscript. All authors approved the final version of the manuscript for submission.

## DISCLOSURE

Dr. Karppinen reports personal fees from lecture fee, Orion Pharma Ltd., personal fees from lecture fee, MSD, personal fees from scientific advisory board, Pfizer, outside the submitted work.

## Data Availability

The data that support the findings of this study are available from the Finnish Student Health Service. Restrictions apply to the availability of these data, which were used under license for this study. Data are available from the authors with the permission of the Finnish Student Health Service.
